# Gender-specific alteration of energy balance and circadian locomotor activity in the *Crtc1* knockout mouse model of depression

**DOI:** 10.1038/s41398-017-0023-4

**Published:** 2017-12-08

**Authors:** Clara Rossetti, Daniel Sciarra, Jean-Marie Petit, Chin B. Eap, Olivier Halfon, Pierre J. Magistretti, Benjamin Boutrel, Jean-René Cardinaux

**Affiliations:** 1 Center for Psychiatric Neuroscience, Department of Psychiatry, University Medical Center, University of Lausanne, Prilly, Switzerland; 2 Service of Child and Adolescent Psychiatry, Department of Psychiatry, University Medical Center, University of Lausanne, Lausanne, Switzerland; 3 Laboratory of Neuroenergetics and Cellular Dynamics, Brain Mind Institute, Ecole Polytechnique Fédérale de Lausanne (EPFL), Lausanne, Switzerland; 4 Unit of Pharmacogenetics and Clinical Psychopharmacology, Center for Psychiatric Neuroscience, Department of Psychiatry, University Medical Center, University of Lausanne, Prilly, Switzerland; 5 School of Pharmaceutical Sciences, University of Geneva, University of Lausanne, Geneva, Switzerland; 6 Division of Biological and Environmental Sciences and Engineering, King Abdullah University of Science and Technology, Thuwal, Saudi Arabia

## Abstract

Obesity and depression are major public health concerns, and there is increasing evidence that they share etiological mechanisms. CREB-regulated transcription coactivator 1 (CRTC1) participates in neurobiological pathways involved in both mood and energy balance regulation. *Crtc1* ^−/−^ mice rapidly develop a depressive-like and obese phenotype in early adulthood, and are therefore a relevant animal model to explore possible common mechanisms underlying mood disorders and obesity. Here, the obese phenotype of male and female *Crtc1* ^−/−^ mice was further characterized by investigating CRTC1’s role in the homeostatic and hedonic regulation of food intake, as well as its influence on daily locomotor activity. *Crtc1* ^−/−^ mice showed a strong gender difference in the homeostatic regulation of energy balance. Mutant males were hyperphagic and rapidly developed obesity on normal chow diet, whereas *Crtc1* ^−/−^ females exhibited mild late-onset obesity without hyperphagia. Overeating of mutant males was accompanied by alterations in the expression of several orexigenic and anorexigenic hypothalamic genes, thus confirming a key role of CRTC1 in the central regulation of food intake. No alteration in preference and conditioned response for saccharine was observed in *Crtc1* ^−^^*/−*^ mice, suggesting that mutant males’ hyperphagia was not due to an altered hedonic regulation of food intake. Intriguingly, mutant males exhibited a hyperphagic behavior only during the resting (diurnal) phase of the light cycle. This abnormal feeding behavior was associated with a higher diurnal locomotor activity indicating that the lack of CRTC1 may affect circadian rhythmicity. Collectively, these findings highlight the male-specific involvement of CRTC1 in the central control of energy balance and circadian locomotor activity.

## Introduction

Obesity, resulting from an impairment of energy balance, represents a main public health concern because of its comorbidity with diabetes, cardiovascular diseases, cancer, and psychiatric disorders^1^. Clinical studies report high prevalence of obesity in patients suffering of chronic mental illnesses, and among depressive subjects, those affected by atypical depression have the strongest odd to develop obesity^2^. If poor nutrition, lack of exercise, and psychiatric medication can explain, at least in part, why this psychiatric population develops obesity, growing evidence supports the hypothesis that the association of obesity and depression may originate from shared biological pathways^3–5^. Hence, in order to improve psychiatric outcomes, a better understanding of the neurobiological adaptations shared by these two pathologies is of the highest importance.

CREB-regulated transcription coactivator 1 (CRTC1) has recently been involved in mood regulation and energy balance^6–10^. This transcription coactivator, together with CRTC2 and CRTC3, constitutes a family of proteins that can detect cellular activation and stimulate CREB-dependent gene expression, independently of CREB phosphorylation^11–13^. CRTCs can sense many external inputs, such as hormones and neurotransmitters, by detecting cytoplasmic increase of calcium and cAMP levels. Upon cellular activation, they become dephosphorylated and translocate to the nucleus where they bind to CREB and activate the transcription of several tissue-specific CREB-regulated genes. Despite their different expression throughout the body, all the three CRTC members are involved in energy metabolism. CRTC3 is essentially expressed in the adipose tissue where it facilitates fat deposition^14^, whereas CRTC2 has been found to regulate gluconeogenesis and insulin sensitivity in the liver and the pancreas, respectively^13,15–17^. More recently, CRTC2 has also been detected in the brain, and in particular in the hypothalamus, where it is able to link glucose sensing with gene regulation^18^. Among the CRTC family members, CRTC1 is the most abundant in the brain, in particular in the prefrontal cortex, the hippocampus, the amygdala, and the hypothalamus^19–21^. Inside the hypothalamus, a key region for regulating energy balance, CRTC1 is present in the arcuate nucleus (ARC), in the ventromedial, and in the paraventricular hypothalamus^21^.

Recent evidence collected in *Crtc1* ^−/−^ mice has established that not only the lack of CRTC1 induces hyperphagic obesity^6,7^, but it also triggers a depressive-like phenotype, which suggests that CRTC1 plays a role in mood disorder etiology and antidepressant response^8–10^. In agreement with these animal studies, two human investigations have highlighted an association of *CRTC1* polymorphisms with body mass index and fat mass, and have suggested that CRTC1 is involved in the high prevalence of overweight and obesity observed in psychiatric patients and in subjects from the general population with major depressive disorder^22,23^. Altogether, these findings suggest that CRTC1 is a transcriptional coactivator reciprocally involved in the bidirectional relation between obesity and depression.

In this study, we further investigated the consequences of the lack of CRTC1 on the energy balance of *Crtc1* ^−/−^ male and female mice, with the aim of better defining CRTC1-regulated molecular pathways involved in obesity, and possibly in mood disorders as well. Alterations in energy intake were assessed using three different approaches: (I) monitoring food consumption and body weight gain, (II) evaluating the expression of multiple genes in the ARC of the hypothalamus and (III) determining the integrity of the hedonic regulation of food intake by testing the preference and the conditioned response for saccharine. Moreover, the influence of CRTC1 on energy expenditure was assessed through the measure of the spontaneous and voluntary locomotor activity. Overall, our results confirm that CRTC1 is critical for the maintenance of energy balance and show, for the first time, the presence of a clear sexual dimorphism in the obesity of *Crtc1* ^−/−^ mice, because males develop a more severe obesity than females, and are more active and hyperphagic during the resting (diurnal) phase of the light/dark cycle.

## Materials and methods

### Mice

*Crtc1* ^−/−^ mice and *Crtc1* ^*+/+*^ wild-type (WT) littermates were obtained and genotyped as previously described^7,8^. Mice were housed in a temperature and humidity-controlled environment, and received water and standard rodent chow ad libitum under a 12-h dark–light cycle, unless otherwise specified. All behavioral experiments were carried out in the dark phase of the light cycle. Body weight measurements and behavioral assessments of the mice were not randomized nor blinded to the investigator. The procedures were performed in conformity with the Swiss National Institutional Guidelines on Animal Experimentation and approved by the Cantonal Veterinary Office.

### Body weight and food intake measurements

Six-week-old males and females were single-housed, and their body weight and food intake measured weekly. Males were divided in two groups; the first was monitored until 8 weeks of age and the second until 36 weeks. Assessment of food intake and body weight of females was protracted until 52 weeks of age. Food consumption during the dark and light phase of the cycle was measured when males and females were 30 weeks old. The development of obesity in mutant females was also studied in an additional group of 6-week-old mice fed ad libitum with a high fat diet (HFD 2127—KLIBA NAFAG: carbohydrates 41.1%, proteins 23.9%, fats 35%, 5.68 Kcal/g) during 50 days.

### Gene expression analysis

After body weight measurements, mice were killed and fresh brains were rapidly sliced into 1 mm-thick coronal sections in a mouse brain stainless steel matrix. The slice containing the hypothalamus was used to dissect the ARC by a micro-punch technique (0.98 mm diameter micro-punch, Stoelting). ARC RNA was extracted with an RNeasy Plus Minikit (Qiagen, Valencia, CA, USA) and converted in cDNA by reverse transcription reaction using TaqMan Reverse Transcriptase Reagents (Applied Biosystem). Real-time PCR amplification was performed with an ABIPRISM 7500 cycler and SYBER green PCR Master Mix (Applied Biosystem) using specific sets of primers (Mycrosynth AG). Forward and reverse primers for the tested genes are the following: *β-actin = *forward: 5′-GCTTCTTTGCAGCTCCTTCGT-3′, reverse: 5′-ATATCGTCATCCATGGCGAAC-3′; *AgRP = *forward: 5′-CGGAGGTGCTAGATCCACAGA-3′, reverse: 5′-AGGACTCGTGCAGCCTTACAC-3′; *Cart* = forward: 5′-TTCCTGCAATTCTTTCCTCTTGA-3′, reverse: 5′-GGGAATATGGGAACCGAAGGT-3′; *Fto = *forward: 5′-GGACATCGAGACACCAGGAT-3′, reverse: 5′-AGGTGCCTGTTGAGCACTCT-3′; *Glp1r = *forward: 5′-ACTTTCTTTCTCCGCCTTGGT-3′, reverse: 5′-TTCCTGGTGCAGTGCAAGTG-3′; *LepRb = *forward: 5′-GCATGCAGAATCAGTGATATTTGG-3′, reverse: 5′-CAAGCTGTATCGACACTGATTTCTTC-3′; *Npy = *forward: 5′-CAGAAAACGCCCCCAGAAC-3′, reverse: 5′-CGGGAGAACAAGTTTCATTTCC-3′; *Npy-rY1* = forward: 5′-CAAGATATACATTCGCTTGA-3′, reverse: 5′-AGATTGTGGTTGCAGG-3′; *Nor1* = forward: 5′-TGGCTCGACTCCATTAAAGAC-3′, reverse: 5′-TGCATAGCTCCTCCACTCTCT-3′. All samples were analyzed in triplicates. Relative gene expression was measured with the comparative ΔΔ*Ct* method^24^ and normalized with *β-actin* transcript levels.

### Preference and conditioned response for saccharine

#### Two-bottle choice test

The preference for saccharine was assessed in *Crtc1* ^−/−^ and WT mice at 16 weeks of age. Individually housed, mice had simultaneously free access to two drinking bottles, one containing tap water and the other 0.2% saccharine solution. The position of the two bottles in the cage was interchanged every day. Water and saccharine consumption was measured daily, along 4 days. Saccharine preference was calculated as preference ratio = (saccharine consumption/total liquid consumption) × 100.

#### Saccharine operant conditioning

Saccharine self-administration was measured in 8-week-old mice using eight operant chambers (Med Associated Inc., St. Albans, VT, USA). Mice were trained to self-administer saccharine 0.2% liquid reward on a fixed ratio 1, time out 3 s (FR1 TO3) schedule of reinforcement in the presence of an olfactory cue (apple aroma, Givaudan, Dübendorf, Switzerland) during 30-min daily sessions. A single nose entry in the active nosepoke activated a liquid dipper equipped with a 0.01 ml cup and a light cue located inside the nosepoke. The liquid reward remained available for 3 s once access to the liquid dipper had been detected with head entry detectors. Supplementary entries in the active nosepoke in the absence of head entry detection above the liquid dipper and entries in the inactive nosepoke were recorded but had no further consequence. During the early phase of acquisition, mice were restricted to the 85% of their daily food consumption. Once fed ad libitum, mice were trained until stable levels of intake (≤25% variation of the mean responses for three consecutive sessions). To reach this stability criterion of the acquisition phase, 18 and 13 daily sessions were required for males and females, respectively. Afterwards, mice underwent an extinction phase, which lasted 7 and 8 daily sessions for males and females, respectively, until completion of the extinction criterion (<30% of the mean responses obtained during the 3 days achieving the stabilization criterion across 3 consecutive extinction sessions). The day after the last extinction session, mice were tested for reinstatement of their nosepoking activities upon presentation of the olfactory and light cues, and the numbers of entries in the active and inactive nosepokes were recorded during 30-min sessions while no liquid reward was delivered. The number of nosepokes was used to compare *Crtc1* ^−/−^ and WT mice along the entire procedure.

### Measures of locomotor activity

#### Spontaneous locomotor activity

Spontaneous activity was recorded in standard conditions (food and water ad lib., L:D of 12:12) with a home-made apparatus (“NEPAAL”^25^). This device consists of 12 transparent Plexiglas home cages (30 × 50 × 40 cm) with sawdust on the floor and equipped with a passive infrared movement detector on the top, a food dispenser and a bottle of water. Eight-week-old mice were individually placed in the activity cages and habituated 1 week to the novel environment before starting recording. Activity was measured by 15 min bins over 7 days and was expressed in arbitrary units.

#### Activity in running wheel

Voluntary wheel running was monitored in standard cages equipped with a stainless steel wheel. Each wheel measured 23 cm of diameter and had a running lane width of 7.5 cm (2B Biological Instruments, Varese, Italy). Animals of 8 weeks of age were individually housed in running wheel cages and acclimated for 7 days before starting measurements. Water and food were provided ad libitum. Running activity (number of wheel revolutions/h) was recorded continuously for 1 week. For each mouse, the activity in the dark and light phase of the cycle was measured as the average of the last 3 days of the test, in which mice performance was stable.

### Statistical analyses

All data reported in the text and figures are means ± s.e.m. The number of independent samples of each group is indicated in the figure legend. The non-homogenous number of animals tested in each group is either due to breeding difficulties (small number of WT and *Crtc1* ^−/−^ mice per litter and limited breeding colony size), to technical problems during the gene expression analyses, or to the exclusion of samples according to a predefined criterion (scores that are more than 2 standard deviations above or below the mean). Sample sizes were determined based on power analysis and common practice in behavioral experiments (~12 animals per group). Before performing statistical comparisons, the normality of data distribution was verified with Shapiro–Wilk test. Since normality of saccharine operant conditioning and spontaneous activity were not fulfilled, raw data underwent logarithmic transformation. Basal body weight, gene expression, saccharine preference, total spontaneous activity, and total food intake over 24 h were compared with Student’s *t* test for independent samples, after evaluation of homoscedasticity with Levene’s test. Statistical analysis of body weight gain, cumulative food intake, saccharine operant conditioning, spontaneous activity, and food consumption in the dark and light phases, was done using a repeated measure two-way ANOVA followed by Bonferroni post hoc test for multiple comparisons, when appropriate. Sphericity assumption, for repeated measures, was verified with Mauchly’s test and degrees of freedom eventually corrected by Greenhouse–Geisser *ε* value. Running activity in dark and light phase was analyzed with Mann–Whitney *U* test for non-parametric independent samples. Statistical power of all analysis was between 0.8 and 1.0. Level of significance was set at *P* ≤ 0.05. All statistical analyses were performed with IBM SPSS Statistic 23.0 software (IBM, Armonk, NY, USA).

## Results

### *Crtc1*^−/−^ male mice are hyperphagic and develop obesity

**Fig. 1:**
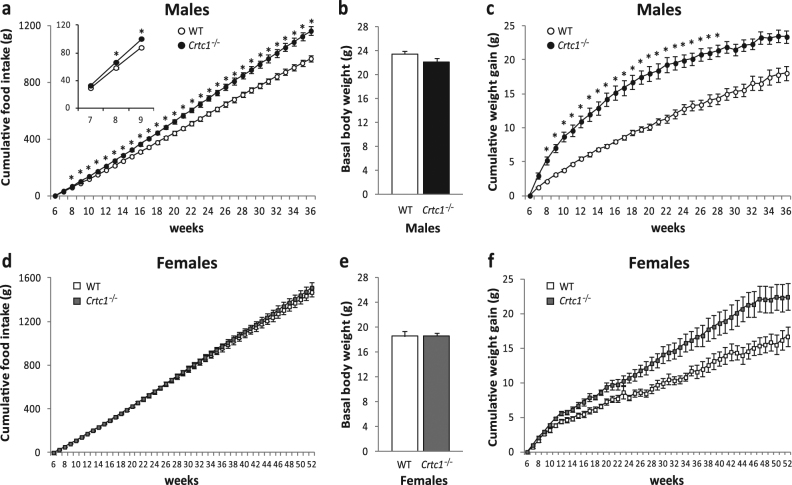
Effect of CRTC1 deficiency on food intake and body weight in male and female mice. **a**–**c** Cumulative food intake and body weight gain in *Crtc1* ^−/−^ (*n* = 12) and wild-type (WT) males (*n* = 14). **a** *Crtc1* ^−/−^ males eat significantly more than WT (repeated measures two-way ANOVA: genotype: *F*_(1,24)_ = 20.737, *P* < 0.001; weeks: *F*_(1.65,39.66)_ = 34,572.49, *P* < 0.001; genotype × weeks: *F*_(1.65,39.66)_ = 3.309, *P* = 0.055) and their overeating is already present at 7 weeks of age, as shown in the inserted graph **b** Comparison of basal body weight in 6-week-old WT and *Crtc1* ^−/−^ males shows no significant difference (*P* = 0.129). **c** *Crtc1* ^−/−^ males gain more weight than WT (repeated measures two-way ANOVA: genotype: *F*_(1, 24)_ = 53.537; *P* < 0.001; weeks: *F*_(3.001,72.031)_ = 513.07; *P* < 0.001; genotype × weeks: *F*_(3.001,72.031)_ = 513.073; *P* < 0.001). **d**–**f** Cumulative food intake and body weight gain in *Crtc1* ^−/−^ (*n* = 11) and WT females (*n* = 15). **d** No hyperphagia was observed in *Crtc1* ^−/−^ females until 52 weeks of age (repeated measures two-way ANOVA: genotype: *F*_(1,24)_ = 0.176, *P* = 0.678; weeks: *F*_(1.041,24.982)_ = 1944.94, *P* < 0.001; genotype × weeks: *F*_(1.041,24.982)_ = 0.43, *P* = 0.526). **e** Comparison of basal body weight in 6 weeks old WT and *Crtc1* ^−/−^ females. Basal body weight was identical in both genotypes (*P* = 0.969). **f** Body weight gain of *Crtc1* ^−/−^ and WT females (repeated measures two-way ANOVA: genotype: *F*_(1,24)_ = 10.527, *P* = 0.034; weeks: *F*_(3.185,76.443)_ = 263.167, *P* < 0.001; genotype × weeks: *F*_(3.185,76.443)_ = 0.508; *P* = 0.689). *P < 0.05 Bonferroni post hoc test

To characterize the obesity of *Crtc1* ^−/−^ mice, we monitored food intake and body weight until 36 weeks of age in males and 52 weeks in females. Measures of food consumption revealed that starting from 8 weeks of age *Crtc1* ^−/−^ males were hyperphagic and ate significantly more than controls (Fig. 1a). Unlike males, females did not exhibit any overeating behavior (Fig. 1d).

No significant difference was found in body weight between young *Crtc1* ^−/−^ and WT mice. Indeed, at 6 weeks of age males (*Crtc1* ^−/−^ = 22.10 ± 0.32, WT = 23.47 ± 0.41) and females (*Crtc1* ^−/−^ = 18.58 ± 0.46, WT = 18.55 ± 0.79) of both genotypes showed similar weight (Fig. 1b, e). However, *Crtc1* ^−/−^ males compared to females, exhibited a stronger body weight gain with aging. According with their early overeating, *Crtc1* ^−/−^ males gained quickly more weight than controls (Fig. 1c). Measures of body weight gain revealed that *Crtc1* ^−/−^ males were overweight from the 8th week of age (Bonferroni post hoc, *P = *0.0019).

In contrast to males, *Crtc1* ^−/−^ females, which did not show overeating, had a slower and more moderate body weight increase (Fig. 1f), as attested by repeated measures two-way ANOVA (genotype: *F*_(1,24)_ = 10.527, *P = *0.034; weeks: *F*_(__3.185,76.443)_ = 263.167, *P* < 0.001; genotype × weeks: *F*_(__3.185,76.443)_ = 0.508; *P* = 0.689), even though the Bonferroni post hoc test did not show any significant comparison. The elevated number of multiple comparisons affected this post hoc test, and therefore it did not reveal specifically at which weeks of age *Crtc1* ^−/−^ females had a significant higher body weight gain, as compared with WT females. Nevertheless, the significant effect of the genotype revealed by the ANOVA showed that mutant females globally gained more weight than WT females. An independent weight gain comparison of the 52-week-old *Crtc1* ^−/−^ and WT females actually confirmed this difference (*Crtc1* ^−/−^ = 22.4 ± 1.9 versus WT = 16.7 ± 1.4, Student’s *t* test, *P* = 0.023). Further corroborating the absence of hyperphagia and early-onset obesity in mutant females, 6-week-old mice were fed ad libitum with HFD for 50 days (Supplementary Fig. S1). *Crtc1* ^−/−^ and WT females consumed equivalent amount of HFD (repeated measures two-way ANOVA: genotype: *F*_(__1,12)_ = 1.637, *P* = 0.225; days: *F*_(__1.03,12.881)_ = 833.32, *P* < 0.001; genotype × days: *F*_(__1.03,12.881)_ = 1.336; *P* = 0.278) and gained similar body weight (repeated measures two-way ANOVA: genotype: *F*_(__1,12)_ = 0.62, *P* = 0.808; days: *F*_(__1.233,14793)_ = 37.464, *P* < 0.001; genotype × days: *F*_(__1.233,14793)_ = 0.421; *P* = 0.568). Taken together, these data show that the lack of CRTC1 seriously increases feeding behavior and body weight gain of male mice, whereas it has only a weak impact on energy balance of mutant females.

### Orexigenic and anorexigenic gene expression is altered in the ARC of *Crtc1*^−/−^ males

**Fig. 2:**
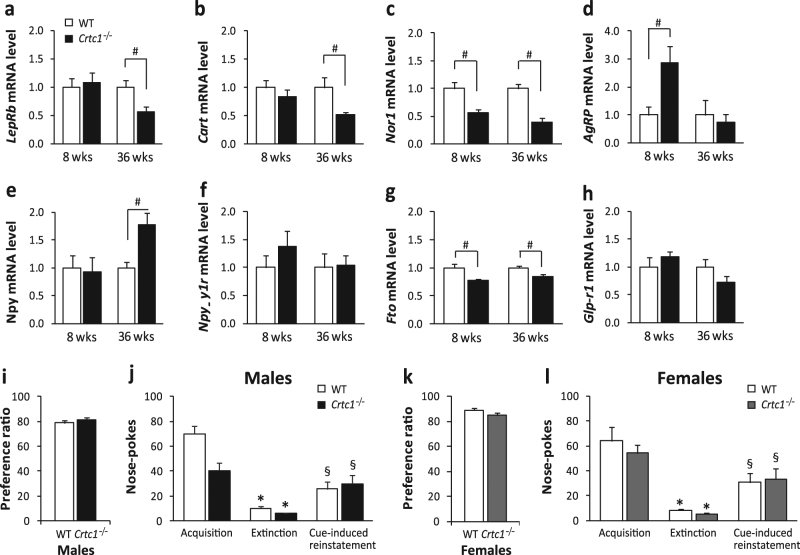
**a**–**h** ARC gene expression in 8-week-old and 36-week-old WT and *Crtc1* ^−/−^ males **a** *LepRb* mRNA levels were comparable in 8-week-old males (WT: *n* = 12, *Crtc1* ^−/−^: *n* = 7), whereas they were reduced in 36-week-old *Crtc1* ^−/−^ males (WT: *n* = 8, *Crtc1* ^−/−^: *n* = 7, *P* = 0.0143). **b** *Cart* expression was similar in the two groups at 8 weeks (WT: *n* = 12, *Crtc1* ^−/−^: *n* = 8), whereas at 36 weeks *Crtc1* ^−/−^ males had reduced *Cart* mRNA compared to WT (WT: *n* = 7, *Crtc1* ^−/−^: *n* = 8, *P* = 0.0294). **c** *Nor1* mRNA level was significantly reduced in *Crtc1* mutant males both at 8 weeks (WT: *n* = 12, *Crtc1* ^−/−^: *n* = 7, *P* = 0.008) and 36 weeks (WT: *n* = 7, *Crtc1* ^−/−^: *n* = 8, *P* < 0.001). **d** *AgRP* transcription was upregulated in 8-week-old *Crtc1* mutant males (WT: *n* = 7, *Crtc1* ^−/−^: *n* = 8, *P* = 0.020) but not in 36-week-old *Crtc1* ^−/−^ males (WT: *n* = 10, *Crtc1* ^−/−^: *n* = 12). **e** *Npy* mRNA levels increased in 36-week-old *Crtc1* mutant mice (WT: *n* = 9, *Crtc1* ^−/−^: *n* = 13, *P* = 0.005), but not in 8-week-old mice (WT: *n* = 12, *Crtc1* ^−/−^: *n* = 8). **f** *Npy-y1* expression was not altered in *Crtc1* mutant males at both ages (8 weeks: WT: *n* = 10, *Crtc1* ^−/−^: *n* = 8; 36 weeks: WT: *n* = 10, *Crtc1* ^−/−^: *n* = 10).**g** *Fto* mRNA level was lowered both in 8-week-old (WT: *n* = 12, *Crtc1* ^−/−^: *n* = 8, *P* = 0.004) and 36-week-old *Crtc1* mutant males (WT: *n* = 8, *Crtc1* ^−/−^: *n* = 8, *P* = 0.019). **h** *Glp-r1* transcript levels were not affected by the lack of CRTC1 (8 weeks: WT: *n* = 12, *Crtc1* ^−/−^: *n* = 7; 36 weeks: WT: *n* = 12, *Crtc1* ^−/−^: *n* = 10). **i**–**l** Preference and conditioned response for 0.2% saccharine solution. **i** Both *Crtc1* ^−/−^ (*n* = 5) and WT males (*n* = 8) showed strong preference for saccharine in a two-bottle choice test. No difference was observed between the two genotypes (*P* = 0.521)**. j** Consumption of saccharine in the operant conditioning paradigm. *Crtc1* ^−/−^ males (*n* = 8) collected less saccharine rewards during the acquisition phase, compared to WT (*n* = 8), although the difference was not statistically different. Similar motivation for saccharine was observed during the cue-induced reinstatement phase (repeated measures two-way ANOVA: genotype: *F*_(1,14)_ = 2.780, *P* = 0.118; phase: *F*_(2,28)_ = 103.44, *P* < 0.001; phase × genotype: *F*_(__2,28)_ = 3.688, *P* = 0.038). **k** *Crtc1* ^−/−^ (*n* = 9) and WT females (*n* = 9) showed equal preference for saccharine in the two-bottle choice test (*P* = 0.246). **l** No statistically significant difference was found in motivated consumption of saccharine between *Crtc1* ^−/−^ (*n* = 12) and WT females (*n* = 13) neither in the acquisition phase nor in the cue-induced reinstatement phase (repeated measures two-way ANOVA: genotype: *F*_(1,23)_ = 0.622, *P* = 0.438; phase: *F*_(2,46)_ = 117.39; *P* < 0.001; phase × genotype: *F*_(2,46)_ = 3.121; *P* = 0.054). ^#^ *P* < 0.05, Student’s *t* test. *P < 0.05 Bonferroni post hoc test vs acquisition. ^§^ *P* < 0.05 Bonferroni post hoc test vs. extinction

Given the presence of CRTC1 in the ARC and the pivotal role played by this structure in the homeostatic regulation of food intake, we investigated whether *Crtc1* ^−/−^ males’ hyperphagia could be linked to an imbalance in the expression of anorexigenic and orexigenic genes. We compared ARC gene expression in 8-week-old and 36-week-old *Crtc1* ^−/−^ and WT males (Table 1 and Fig. 2a–h), as well as the relative expression of four anorexigenic genes (*LepRb*, *Cart*, *Nor1*, and *Glp-r1*), three orexigenic genes (*AgRP*, *Npy*, and *Npy-y1*) and the fat-mass-related and obesity-related gene (*Fto*). In the ARC of 36-week-old *Crtc1* ^−/−^ males, we found significant downregulation of *LepRb*, *Cart*, *Nor1*, and *Fto*, increased expression of *Npy* and no change in the expression of *AgRP*, *Npy-y1*, and *Glp-r1*. At 8 weeks of age, the lack of *Crtc1* induced significant reduction of *Nor1* and *Fto* and upregulation of *AgRP*, as compared with WT males. On the other hand, we did not observe, at this age, any difference in the relative level of *LepRb*, *Cart*, *Npy*, *Npy-y1*, and *Glp-r1*. Consistent with the absence of hyperphagia, 52-week-old mutant females did not exhibit any change of *Cart*, *AgRP*, *Npy*, and *Glp-r1* expression, but only increased levels of *Npy-y1* (Supplementary Table S1 and Supplementary Fig. S2). Collectively, these results show that *Crtc1* ^−/−^ males present perturbed expression of both orexigenic and anorexigenic genes, whereas the lack of CRTC1 does not induce major alterations in the homeostatic regulation of food intake in mutant females.

**Table 1:** Relative mRNA levels of orexigenic and anorexigenic genes in *Crtc1* ^−/−^ and WT males at 8 and 36 weeks of age

	8-week-old male mice			36-week-old male mice		
Gene	WT	*Crtc1* mice	*P* value	WT	*Crtc1* mice	*P* value
*LepRb*	1.000 ± 0.157	1.070 ± 0.187	0.778	1.000 ± 0.119	0.564 ± 0.092	0.014*
*Cart*	1.000 ± 0.129	0.839 ± 0.127	0.408	1.000 ± 0.169	0.521 ± 0.032	0.029*
*Nor1*	1.000 ± 0.107	0.554 ± 0.063	0.008*	1.000 ± 0.085	0.382 ± 0.079	<0.001*
*AgRP*	1.000 ± 0.299	2.851 ± 0.599	0.020*	1.000 ± 0.543	0.732 ± 0.287	0.978
*Npy*	1.000 ± 0.217	0.918 ± 0.268	0.814	1.000 ± 0.109	1.774 ± 0.216	0.005*
*Npy-y1r*	1.000 ± 0.210	1.371 ± 0.282	0.297	1.000 ± 0.256	1.031 ± 0.192	0.923
*Fto*	1.000 ± 0.061	0.767 ± 0.037	0.004*	1.000 ± 0.038	0.844 ± 0.045	0.019*
*Glp-r1*	1.000 ± 0.172	1.178 ± 0.104	0.388	1.000 ± 0.138	0.722 ± 0.106	0.397

*P < 0.05 shown in bold, Student’s *t* test

### The hyperphagia of *Crtc1*^−/−^ males does not depend on increased preference for food rewards

Motivation to consume highly palatable food, which relies on the integrity of the reward system, is also essential for a proper feeding behavior. *Crtc1* is also expressed in brain structures belonging to the reward system, but so far, no study has established whether the functionality of this system is compromised in *Crtc1* ^−/−^ mice. Therefore, we compared *Crtc1* ^−/−^ and WT mice for saccharine preference in a two-bottle choice test and assessed their capacity to seek for saccharine reward in an operant conditioning test.

The two-bottle choice tests revealed that mutant mice and controls had similar preference for saccharine (Fig. 2i, k), with a preference ratio for saccharine of 78.7% ± 2.5 in WT and of 81.2% ± 2.4 in *Crtc1* ^*‒/‒*^ males. As expected, females of both genotype exhibited a clear-cut preference for saccharine as well (WT = 88.9% ± 1.4, *Crtc1* ^*‒/‒*^ = 85.4% ± 2.5).

Both WT and *Crtc1* ^*‒/‒*^ males were then trained for self-administering saccharine in a fixed ratio 1 schedule until stable intake, and after a period of extinction, we assessed their ability to reinstate their saccharine seeking behavior upon presentation of the light and olfactory cues (Fig. 2j). A two-way repeated measure ANOVA revealed a significant main effect for phase (*F*_(2,28)_ = 103.44, *P* < 0.001) and genotype × phase (*F*_(2,28)_ = 3.688, *P* = 0.038), but no effect for genotype (*F*_(__1,14)_ = 2.780, *P* = 0.118). Although Bonferroni post hoc tests did not reveal any significant difference between genotypes, the significant interaction revealed by the ANOVA most likely suggests that mutant males may display a tendency to collect less rewards than controls (WT = 70.0 ± 6.45 versus *Crtc1* ^*‒/‒*^ = 40.5 ± 6.27 active nosepokes). As a consequence, despite both groups of mice exhibited a significant reinstatement of previously extinguished nosepoking behavior (WT = 25.9 ± 5.68 and *Crtc1* ^*‒/‒*^ = 30.0 ± 7.20 active nosepokes), *Crtc1* ^*‒/‒*^ mice manifested an enhanced reinstatement when expressed in function of their baseline intake of saccharine (WT = 36.8% ± 6.3 versus *Crtc1* ^*‒/‒*^ = 71.0% ± 12.3, Student’s *t* test, *P* = 0.026), most likely due to a ceiling effect observed during saccharine consumption. In contrast, mutant and control females both exhibited similar acquisition, extinction, and reinstatement of saccharine seeking behaviors (Fig. 2l), suggesting that, overall, the lack of CRTC1 most likely does not impair operant responding for saccharine reward in mice.

### *Crtc1*^*−/−*^ male mice move more during the light phase of the cycle

**Fig. 3:**
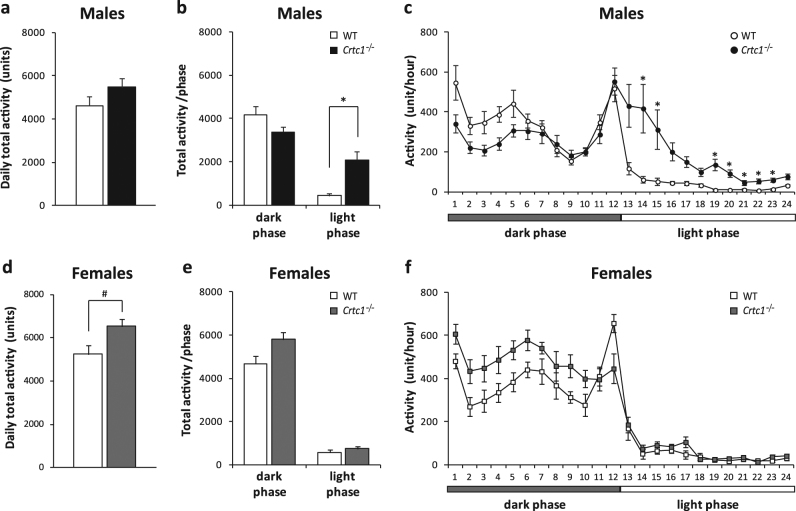
Spontaneous locomotor activity of *Crtc1* mutant mice. **a**–**c** Spontaneous locomotor activity of 9-week-old *Crtc1* ^*−/−*^ (*n* = 12) and WT males (*n* = 12). **a** Total daily spontaneous locomotor activity of *Crtc1* ^*−/−*^ males was similar to that of WT (*P* = 0.162). **b** Spontaneous activity of *Crtc1* ^*−/−*^ and WT males during the dark and light phase. Mutant mice showed slightly reduced activity during the dark phase and increased activity during the light phase (repeated measures two-way ANOVA: genotype: *F*_(1,22)_ = 71.175, *P* < 0.001; phase: *F*_(1,22)_ = 64.713, *P* < 0.001; phase × genotype: *F*_(1,22)_ = 108.43, *P* < 0.001). **c** *Crtc1* ^*−/−*^ and WT daily activity plotted hour-by-hour (repeated measures two-way ANOVA: genotype: *F*_(1,22)_ = 21.029; *P* < 0.001; hours: *F*_(6.65,146.3)_ = 44.42; *P* < 0.001; hours × genotype: *F*_(6.65,146.3)_ = 7.85; *P* < 0.001). **d**–**f** Spontaneous locomotor activity of 9-week-old *Crtc1* ^*−/−*^ (*n* = 11) and WT females (*n* = 9). **d** *Crtc1* ^*−/−*^ females showed significantly higher daily activity than WT (*P* = 0.026). **e** *Crtc1* ^*−/−*^ females moved slightly more than control females in the dark phase (repeated measures two-way ANOVA: genotype: *F*_(1,18)_ = 5.17; *P* = 0.035; phase: *F*_(1,18)_ = 344.64; *P* < 0.001; phase × genotype: *F*_(1,18)_ = 0.56; *P* = 0.464). **f** Daily activity of *Crtc1* ^*−/−*^ and WT females plotted hour-by-hour (repeated measures two-way ANOVA: genotype: *F*_(1,18)_ = 6.017; *P* = 0.025; hours: *F*_(7.068,127.23)_ = 58.55; *P* < 0.001; hours × genotype: *F*_(7.068,127.23)_ = 1.411; *P* = 0.206) *P < 0.05, Bonferroni post hoc test. ^#^ *P* < 0.05, Student’s *t* test

Since reduced energy expenditure could induce body weight gain, we monitored spontaneous locomotor activity of *Crtc1* ^*−/−*^ mice in activity cages along 7 days. The total daily activity of mutant males (Fig. 3a) was similar to that of controls (WT = 4613.80 ± 418.38, *Crtc1* ^*‒/‒*^ = 5465.70 ± 414.35), whereas *Crtc1* ^*‒/‒*^ females (Fig. 3d) exhibited significant higher activity as compared to WT littermates (WT = 5245.10 ± 389.40, *Crtc1* ^*‒/*‒^ = 6535.40 ± 414.35, Student’s *t* test, *P* = 0.026). A deeper analysis of *Crtc1* ^*‒/‒*^ mice activity during the light and dark phase of the light cycle pointed out a strong difference in males’ and females’ behavior (Fig. 3b, e). Mutant females behaved like control females in the light phase and moved a little bit more during the dark phase as shown by repeated measures two-way ANOVA. (genotype: *F*_(1,18)_ = 5.17; *P* = 0.035; phase: *F*_(1,18)_ = 344.64; *P* < 0.001; phase × genotype: *F*_(1,18)_ = 0.56; *P* = 0.464). However, Bonferroni post hoc test did not show any statistical difference between WT and mutant females in this phase of the cycle (*P* = 0.237). Intriguingly, *Crtc1* ^*‒/‒*^ males presented opposite behavior moving as much as controls in the dark phase and significantly more in the light phase (Bonferroni post hoc test, *P* < 0.001).

**Fig. 4:**
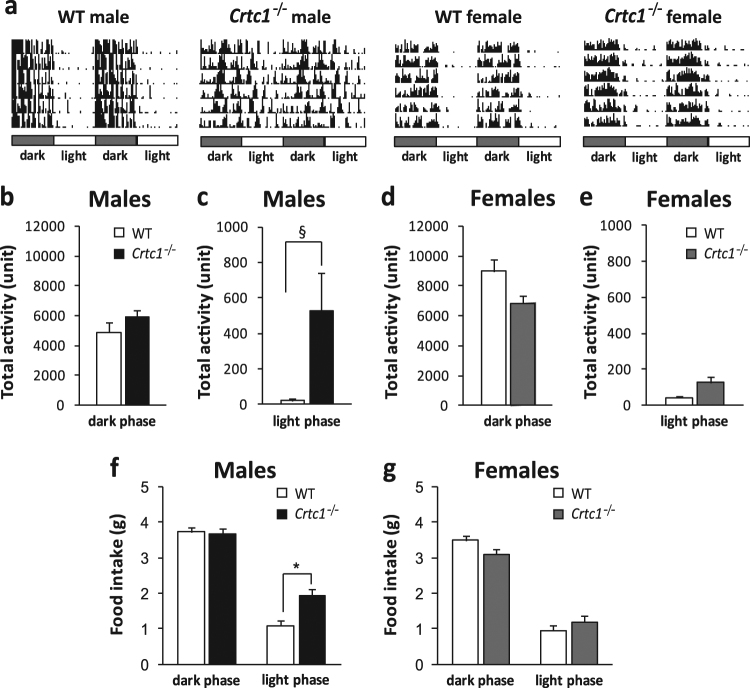
**a** Representative actograms of one *Crtc1* mutant and WT male and female mouse over 7 days. Dark phase and light phase are indicated by the black and the white bars, respectively on the top of each actogram. Each line of the actogram shows the activity of 48 h. **b**–**c** Motivated wheel-running of 9-week-old *Crtc1* ^*−/−*^ (*n* = 11) and WT males (*n* = 9): **b** Total activity in the dark (*P* = 0.002) and **c** in the light phase (*P* = 0.449). **d**–**e** Motivated wheel-running of 9-week-old *Crtc1* ^*−/−*^ (*n* = 12) and WT females (*n* = 13): **d** Total activity in the dark (*P* = 0.077) and **e** in the light phase (*P* = 0.087). **f**–**g** Food consumption of 30-week-old *Crtc1* deficient mice in the dark and light phase of the cycle: **f** *Crtc1* ^*−/−*^ males ate significantly more during the light phase (repeated measures two-way ANOVA: genotype: *F*_(1,19)_ = 10.90; *P* = 0.0037; phase: *F*_(1,19)_ = 123.63; *P* < 0.001; phase × genotype: *F*_(1,19)_ = 5.32; *P* = 0.032). **g** *Crtc1* ^*−/−*^ and control females consumed similar amount of food in both phases (repeated measures two-way ANOVA: genotype: *F*_(1,18)_ = 0.278; *P* = 0.604; phase: *F*_(1,18)_ = 145.23; *P* < 0.001; phase × genotype: *F*_(1,18)_ = 2.95; *P* = 0.102). ^§^ *P* < 0.05, Mann–Whitney *U* test. *P < 0.05, Bonferroni post hoc test

To better characterize this sustained activity of mutant males during the resting phase, we plotted the daily activity on a scale of one-hour intervals (Fig. 3c). Mutant males, compared to controls, moved more when the light was switched on and maintained a significant higher activity along all the light phase (repeated measures two-way ANOVA: genotype: *F*_(1,22)_ = 21.029; *P* < 0.001; hours: *F*_(6.65,146.3)_ = 44.42; *P* < 0.001; hours × genotype: *F*_(6.65,146.3)_ = 7.85; *P* < 0.001). In contrast to what was observed with males, the same hour-by-hour analysis did not reveal any increase in activity of mutant females during the resting phase (Fig. 3f). Seven-day-double-plotted actograms of a representative male and female mouse of both groups clearly showed that *Crtc1* ^*‒/‒*^ males, but not females, have a perturbed circadian locomotor activity (Fig. 4a).

To confirm the increased mobility of *Crtc1* ^*‒/‒*^ males during the light period of the cycle we also measured their voluntary activity in cages equipped with a running wheel (Fig. 4b–e). According to what was observed in the activity cages, mutant males ran more during the light phase, whereas their wheel turns in the dark phase were comparable to those of controls. Consistent with their spontaneous activity, mutant females (Fig. 4d, e) ran as much as control females during the light phase. Taken together, these results show that mutant males, but not females, move more in the resting phase and have a perturbed light/dark activity.

### *Crtc1*^−/−^ male mice eat more during the light phase

To verify whether there was a relation between hyperphagia and light phase activity of *Crtc1* ^*‒/‒*^ males, we determined the quantity of food eaten at the end of the dark and the light phase of the daily cycle. 24-hour food intake of *Crtc1* ^*‒/‒*^ males was significantly higher than controls (WT = 4.35 ± 0.15, *Crtc1* ^*‒/‒*^ = 5.60 ± 0.17, *P* = 0.004, Student’s *t* test) confirming the overeating behavior observed during weekly measurements. When we calculated the quantity of food ingested per phase, we found a consumption of 3.66 g ± 0.14 for *Crtc1* ^*‒/‒*^ males and of 3.36 g ± 0.13 for WT in the dark phase and of 1.93 g ± 0.19 and 1.00 g ± 0.14 (Bonferroni post hoc test, *P* < 0.001) in the light phase, respectively (Fig. 4f). These data support our hypothesis that the hyperphagia of *Crtc1* ^*‒/‒*^ males occurs only during the light phase of the cycle. As expected, *Crtc1* ^*‒/‒*^ females (Fig. 4g), which did not show overeating along all measures of food intake, ate as much as their WT littermates in 24-h (WT = 4.44 g ± 0.14, *Crtc1* ^*‒/‒*^ = 4.28 g ± 0.24). Food consumption in the active phase (WT = 3.49 g ± 0.13, *Crtc1* ^*‒/‒*^ = 3.09 g ± 0.12) and in the resting phase (WT = 0.95 g ± 0.11, *Crtc1* ^*‒/‒*.^ = 1.19 g ± 0.17) was similar in both genotypes. In conclusion, these data suggest that the obesity of *Crtc1* mutant males depends, at least in part, on increased food consumption during the resting phase.

## Discussion

Overall, our work confirms the crucial role played by CRTC1 in energy balance, previously observed by Altarejos et al.^6^, and shows that male mice lacking CRTC1 are hyperphagic and more vulnerable to develop obesity than mutant females, and this, from the beginning of adulthood. Indeed, mutant females exhibited a mild late-onset obesity without hyperphagia. We also found similar preference and self-administration responding for saccharine in both sexes. Finally, we observed an altered circadian activity in mutant males only.

So far, many neurobiological pathways responsible for food intake regulation have been identified in the brain^26^. The hypothalamus plays an essential role in controlling hunger and satiety. Due to the anatomical localization of the ARC, it is the first hypothalamic region that receives peripheral signals informing the brain about energy availability. Among these peripheral signals, leptin that is released by adipocytes, promotes fasting by stimulating CART/POMC neurons and inhibiting AgRP/NPY neurons through the binding to its receptor (LepRb)^27^. However, in obesity the large amount of leptin released by the adipose tissue leads to ARC neurons leptin insensitivity (also called leptin resistance) and contributes to food overconsumption. Interestingly, Altarejos et al. have observed that 36-week-old *Crtc1* ^*‒/‒*^ mice are leptin resistant^6^. Many mechanisms have been proposed to explain leptin resistance, and one of them is a weak expression of the long form of the leptin receptors (LepRb) in the brain^28^. Accordingly, we found a significant reduction of the transcript levels for *LepRb* in overweight *Crtc1* ^*‒/‒*^ males, but not in young mutant males.

Recent studies have also shown that leptin can modulate the transcription of CREB-dependent genes by directly facilitating CREB phosphorylation^29,30^ or by enhancing CRTC1 nuclear translocation^31^. Because *Cart* is a CREB-regulated gene, we expected to find reduced levels of *Cart* transcript in both young and old *Crtc1* ^*‒/‒*^ males. Surprisingly, only old and obese *Crtc1* ^*‒/‒*^ males have lowered levels of *Cart* transcript, whereas young mice have not. Therefore, the deletion of *Crtc1* per se does not seem to affect *Cart* transcription. Two mechanisms may explain this observation. First, our mice are constitutive knockout and the presence of CRTC2 in the ARC might compensate for CRTC1 deficiency^18^. Second, CREB phosphorylation is independent from CRTC1 activity and so, even in its absence, leptin-induced CREB activation may be sufficient to allow *Cart* transcription. Conversely, the impairment of leptin signal in old *Crtc1* ^*‒/‒*^ males, due to lowered *LepRb* expression, would have a more detrimental effect on CREB activation leading to reduced *Cart* transcription. Together, these results suggest that appropriate leptin signaling may be crucial for *Cart* expression.

Since young *Crtc1* ^*‒/‒*^ males, despite unchanged *Cart* expression, ate more than their age-matched controls, we explored the possibility that *Crtc1* ^*‒/‒*^ males overeating would depend on the impaired expression of other CREB-regulated genes. The neuron-derived orphan receptor 1 (NOR1), also known as NR4A3, is a protein belonging to the nuclear receptor superfamily of transcription factors^32^. The transcription of *Nor1* is under the control of CREB and we already reported lowered *Nor1* mRNA levels in the prefrontal cortex and in the hippocampus of *Crtc1* ^*‒/‒*^ mice^8^. Recently, a work of Kim et al. has shown that, in ARC neurons leptin seems to facilitate *Nor1* transcription through CREB recruitment and that in turn, *Nor1* would reduce appetite by antagonizing glucocorticoid-induced AgRP release and inhibiting *Npy* transcription^33^. According to this mechanism, we found that *Crtc1* ^*‒/‒*^ males, compared to their age-matched controls, have significantly lower levels of *Nor1* transcript. Moreover, this change in *Nor1* expression was associated with a rise of *AgRP and Npy* mRNA level in young and old *Crtc1* ^*‒/‒*^ males, respectively. Overall, our findings suggest that, the overeating of young *Crtc1* ^*‒/‒*^ males may be induced by the impaired expression of *Nor1* and that the appetite of older *Crtc1* ^*‒/‒*^ mice would be affected by a concurrent reduction of *Nor1* and *Cart* transcription.

The gene coding for the NPY receptor type 1 (NPY-1R) is another anorexic CREB-regulated gene^34^. The comparison between *Crtc1* mutant males and WT littermates at 8 and 36 weeks of age did not reveal any alteration in the expression of this gene indicating that it would not play a critical role in their hyperphagic behavior.

Fat-mass-related and obesity-related gene (*Fto*) is an mRNA demethylase whose enzymatic activity has been associated with increased body mass index and obesity vulnerability. Although its physiological functions remain largely unknown, recent investigations have begun to unravel the link between this enzyme and energy balance^35^. In rodents, it has been found that ARC *Fto* expression is lowered during fasting^36^. Moreover, it seems that this enzyme would delay CREB dephosphorylation and would inhibit food intake through the expression of *Npy-1r* and *Bdnf* ^37^. In contrast to these previous works, we found a lower level of *Fto* transcript in both young and old *Crtc1* ^*‒/‒*^ males. Therefore, additional studies are required to understand more in detail the physiological role of FTO in energy balance regulation, and how CRTC1 interferes with the transcription of this mRNA demethylase.

The glucagon-like peptide 1 is a peptide that acts as a strong feeding suppressor in the hypothalamus upon the binding with its receptor (GLP-R1)^38^. The overlapping distribution of GLP-1R and CRTC1 in the hypothalamus, led us to investigate whether the lack of CRTC1 could affect the expression of this receptor, but our findings indeed show no alteration of its expression.

Unlike males, *Crtc1* ^*‒/‒*^ females do not exhibit any food overconsumption. Consistent with a normal feeding behavior, 52-week-old *Crtc1* ^*‒/‒*^ females do not show any modification in the gene expression pattern, except for a significant increase in *Npy-1r* transcript. This sexual dimorphism was not described in the previous study of Altarejos et al^6^. Further investigations are needed to understand why the lack of *Crtc1* impairs males preferentially, and in particular delineating a putative effect of sex hormones on the CREB–CRTC1 pathway would be of the highest relevance. Indeed, Choong et al. established that the vulnerability to develop obesity in patients bearing a CRTC1 polymorphism varied between men and women, suggesting that estrogen levels most likely modulate the effect of this CRTC1 polymorphism on fat accumulation^22^.

Eating is a complex behavior driven by energy need and food rewarding properties. Whereas the homeostatic control of food intake depends on the functionality of the hypothalamic nuclei, the motivation to consume highly palatable food is governed by the reward system. Neurons of both systems are intimately interconnected^39^. In particular, lateral hypothalamic neurons, which integrate signals coming from other hypothalamic nuclei, project to VTA-dopaminergic neurons and regulate reward seeking behavior through the modulation of dopamine release. Leptin participates in this modulation by inhibiting dopamine release and consequently the rewarding properties of food^40^. Thus, impaired leptin signaling, as that observed in case of leptin insensitivity, may facilitate food overconsumption.

Considering the relevance of non-homeostatic feeding^27^, the fact that CRTC1 is abundantly expressed in brain structures belonging to the reward system, and finally the leptin resistance showed by *Crtc1* mutant mice, we explored the possibility that their overeating could arise from impairments in reward appreciation. Noteworthy, regardless of their genotype and sex, all mice exhibited a drastic preference for sweetened taste over tap water, and the ability to acquire a stable saccharine intake in an operant conditioning paradigm. Furthermore, they all manifested a strong capacity to associate reward delivery and reward-paired cues, as attested by the reinstatement of their previously extinguished nosepoking behavior upon presentation of the olfactory and light cues.

Because *Crtc1* ^*‒/‒*^ males may have earned less rewards during acquisition of saccharine self-administration, an observation in line with a previous report of ours indicating that these mutant mice moved less in response to moderate stressful environments^8^, we considered the possibility that *Crtc1* ^*‒/‒*^ males obesity could also depend on insufficient energy expenditure. To answer this question, and avoid confusion due to excessive overweight, we monitored spontaneous and motivated locomotor activity in young *Crtc1* ^*‒/‒*^ mice. Although *Crtc1* ^*‒/‒*^ and WT males had similar total activity, a more detailed analysis of their behavior showed a great difference between the dark and the light phase of the cycle. In fact, mutant males, as expected moved more during the light phase. Contrastingly, *Crtc1* ^*‒/‒*^ females were globally more active than control females but this extra activity was present only in the dark phase. Voluntary exercise in running wheels confirmed that *Crtc1* ^*‒/‒*^ males, but not females, run more than controls in the light phase. Collectively, these results highlight a different locomotor phenotype in *Crtc1* ^*‒/‒*^ females and males and show that these latter exhibit a perturbed nychtemeral activity.

Because of this observation, we tested the hypothesis that diurnal activity of *Crtc1* ^*‒/‒*^ males could coincide with increased food seeking and food intake. Therefore, we measured food intake in the two phases of the light cycle. *Crtc1* ^*‒/‒*^ and WT females consumed similar amount of food both in the dark and light phase. In contrast, mutant males showed overeating only during the resting phase. Altogether, these findings highlight a perturbation of the circadian rhythms of feeding and locomotor activities in *Crtc1* ^*‒/‒*^ males, thus suggesting that *Crtc1* inactivation induces a gender-specific alteration of important functions regulated by the circadian clock network. Interestingly, recent reports have pointed out the participation of CRTC1 in master clock entrainment^41,42^. Future investigations should unravel the role of this transcription coactivator in the regulation of clock genes in the suprachiasmatic nucleus and other brain regions.

Accumulating evidence indicates that disrupted synchronization of feeding and sleeping time with the master clock results in dampening of metabolic and endocrine functions. Concerning obesity, alterations in adipogenesis, satiety signaling, and energy metabolism have all been associated with circadian regulation^43^. Likewise, the lack of synchrony of the internal clock with the daily light cycle affect glucocorticoid levels and stress reactivity facilitating the development of emotional disturbances, namely depression^44^. In conclusion, improved knowledge of the mechanisms through which CRTC1 synchronizes metabolic functions with the light cycle may facilitate our understanding of the biological processes underlying the interaction between obesity and depression.

## Electronic supplementary material

Suppl Table S1

Suppl Figure S1

Suppl Figure S2
