# Supplementary material for: Gender-specific alteration of energy balance and circadian locomotor activity in the Crtc1 knockout mouse model of depression
Source: Transl Psychiatry. 2017 Dec 8;7:1269. doi: 10.1038/s41398-017-0023-4 (PMC5802703; doi:10.1038/s41398-017-0023-4)
Supplement: Supplementary file 1 — Suppl Table S1 [file 41398_2017_23_MOESM1_ESM.docx]

**Table S1**

|  | 52-week-old female mice | | |
| --- | --- | --- | --- |
| Gene | WT | *Crtc1^‒/‒^* mice | P value |
| *Cart* | 1.000 ± 0.125 | 0.987 ± 0.205 | 0.959 |
| *AgRP* | 1.000 ± 0.269 | 0.934 ± 0.247 | 0.862 |
| *Npy* | 1.000 ± 0.224 | 0.703 ± 0.155 | 0.284 |
| *Npy-y1r* | 1.000 ± 0.201 | 1.830 ± 0.226 | **0.018*** |
| *Glp-r1* | 1.000 ± 0.055 | 1.030 ± 0.091 | 0.783 |
